# Personality Traits of Postmenopausal Women with Sexual Dysfunction

**DOI:** 10.1055/s-0038-1673425

**Published:** 2018-10-11

**Authors:** Cristiano Santos de Caires, Marcelo Luis Steiner, Luciano de Melo Pompei, Rodolfo Strufaldi, Cesar Eduardo Fernandes

**Affiliations:** 1 Faculdade de Medicina do ABC, Santo André, SP, Brazil

**Keywords:** Sexual Dysfunction, personality, women's health, postmenopausal, FPI-II, disfunção sexual, personalidade, saúde da mulher, pós-menopausa, IFP-II

## Abstract

**Objective** The aim of the present study is to identify the association between personality traits of postmenopausal women and the occurrence of sexual dysfunction.

**Methods** A total of 43 postmenopausal women were evaluated according to their self-perception of the quality of their sexual life. They answered the following questionnaires: Sociodemographic Profile Questionnaire, Female Sexual Function Index (FSFI), Beck Depression Inventory (BDI) and Factorial Personality Inventory (FPI-II).

**Results** Women with poorer sexual self-perception showed low affective need (*p* < 0.01) and low need for organization (*p* < 0.01). Based on the need for control and opposition, there was no difference between the groups. Groups separated by the scores obtained on the FSFI showed no significant differences.

**Conclusion** Postmenopausal women with lower schooling and personality characteristics that demonstrate low affective and organizational needs are more likely to present sexual dysfunction.

## Abstract (pt)

**Resumo:**

**Objetivo** O objetivo do presente estudo foi identificar a associação entre características de personalidade de mulheres na pós-menopausa e ocorrência de disfunção sexual.

**Método** No total, 43 mulheres na pós-menopausa foram avaliadas de acordo com suas percepções da qualidade de suas vidas sexuais. Elas responderam aos seguintes questionários: Perfil Sociodemográfico, Índice de Função Sexual Feminino (FSFI, na sigla em inglês), Inventário de Depressão Beck (BDI, na sigla em inglês) e Inventário Fatorial de Personalidade (IFP-II).

**Resultados** Mulheres com pior percepção sexual demonstraram baixa necessidade afetiva (*p* < 0,01) e baixa necessidade de organização (*p* < 0,01). Com base na necessidade de controle e oposição, não houve diferença entre os grupos. Os grupos de mulheres separadas pelo FSFI não demonstraram diferenças significativas.

**Conclusão** Mulheres na pós-menopausa com menor escolaridade e características de personalidade que demonstre baixa necessidade afetiva e de organização possuem maior chance de apresentar disfunção sexual.

## Introduction

The menopausal transition is a brief period of time in the circle of life of women that is characterized by metabolic, psychological, or social alterations. The resulting symptoms are: hot flashes, joint pain, mood and sleep disorders, irritability, fatigue, nocturnal sweating, vaginal dryness, generalized anxiety, depression, decreased libido, and manifestation of a decline in sexual life.^1^

Pertaining to the prevalence of female sexual dysfunction, in the year of 2012, Ambler et al^2^ estimated that between 68 and 86.5% of postmenopausal women presented with some kind of sexual problem. In 2010, Nappi et al^3^ found that the decrease in female sexual desire is associated with personal and interpersonal difficulties.

The biological and psychosocial well-being of the woman impacts the sexual response of perimenopausal and postmenopausal women. In 2011, Bal et al^4^ highlighted the importance of personality traits in the quality of life and in the sexuality of postmenopausal women. Few scientific studies have investigated the psychological aspects linked to the sexual experience of postmenopausal women, as well as possible sexual dysfunctions that may occur in this period of life. Nevertheless, in 2007, Pimenta et al^1^ pointed to some psychological factors associated with complaints of sexual dysfunction.

The term ‘personality’ is described by multiple theories in different psychological disciplines. However, a clear definition describes it as an organizing agent, governor of the individual, whose reflection is a series of events that covers all of a person's life.^5^ One meta-analysis on personality disorders in women > 50 years old found that 20% of the studied population are diagnosed with some kind of personality disorder, suggesting an elevated rate of non-adaptive personality functioning in the aging process.^6^

Therefore, it becomes interesting to observe if specific traits of the personalities of postmenopausal women are related to female sexual disorders. According to the aforementioned information, our objective is to identify if the personality traits of postmenopausal women are associated with the incidence of sexual dysfunction.

## Methods

From January to July 2014, 200 female patients who took medical routine exams at the climacteric ambulatory of the Centro de Atenção Integral à Saúde da Mulher of São Bernardo do Campo (CAISM-SBC, in the Portuguese acronym) were invited to participate as subjects of the present study. This study was approved by the ethical committee in medical research of the Faculdade de Medicina do ABC (FMABC, in the Portuguese acronym).

As requirements to participate in the present study, women should be on the postmenopausal period (at least 12 months in amenorrhea), aged between 40 and 65 years old, have a love partner without sexual complaints, agree and sign an informed consent form. Subjects were excluded if they had entered the menopausal period by surgical intervention, with dyspareunia, compromising physical limitation to sexual activity, vaginismus, chronic pelvic pain, diabetes, arterial hypertension, or were users of estrogen and androgen replacement therapy and of antidepressant drugs.

### Procedures

It is important to highlight that all subjects were invited to participate in the present research while waiting for medical care. Those who accepted to participate were taken to a private room and, once it was determined that they fulfilled the eligibility criteria, answered the questionnaires.

According to the methodology used by Davis et al,^7^ the patients were divided into two groups, after answering the following questions:

a) Before menopause, could you say that your sexual life was good or satisfactory?
b) Since menopause, did you have an important decrease in your sexual desire?
c) Since menopause, has your sexual activity decreased?
d) Are you worried or unsatisfied with your current sexual desire or interest?
e) Do you wish to elevate your level of interest or desire for sex and for your sexual activity?

The subjects who answered affirmatively to all five questions were characterized as women with poor sexual self-perception while the others as good sexual self-perception. Previous studies presented a similar methodology.^7^ ^8^ ^9^

The included subjects were submitted to the following instruments: the sociodemographic profile questionnaire; the factorial personality inventory II (FPI-II);^10^ the beck depression inventory (BDI);^11^ and the female sexual function index (FSFI). Both the questionnaires and the inventories are self-administered, yet the same assessor was present in the whole data extraction phase to solve any doubts.

First of all, the Sociodemographic Profile Questionnaire was filled out, followed by the BDI; then, the FSFI was applied, and the FPI-II was filled out. The tests were filled out on an average of 45 minutes. The data of the questionnaire was divided as follows:

**Sociodemographic Profile Questionnaire**: date of birth, age, years of study, profession, occupation and marital status.
**Factorial Personality Inventory (2013):** self-administered personality inventory developed to evaluate personality traits or basic needs of a person or a group of people. Composed by a hundred items, each assessed with a *Likert* scale varying from 1 (non-characteristic) to 7 (totally characteristic), it evaluates thirteen characteristics/needs or motives (psychological), setting three groups of second order:
  1. Affective necessity (affiliation, assistance, gentle, intraception, deference, and change).
  2. Organization necessity (order, performance, and persistency).
  3. Control and opposition necessity (exhibition, aggression, autonomy, and dominance).
  According to Peres et al,^12^ the FPI “has adequate psychometric properties, since it was validated and standardized to the Brazilian population.” In the data analysis, the similar percentile will be verified, established by the proper instrument manual. The FPI considers important personality traits scores below the percentile of 30 and above the percentile of 70.
  **Beck Depression Inventory:**^11^ composed by self-administered 21 items, with scales which vary from 0 to 3, according to the affirmatives in each question. The higher the score, the higher the prevalence of depressive symptoms will be. The symptoms classification levels follow the scores: minimum (0 to 11 points), mild (12 to 19 points), moderate (20 to 35 points) and severe (36 to 63 points).
  **Female Sexual Function Index:** the questionnaire comprises nineteen items, whose objective is to evaluate the female sexual functioning in six fields, which offers a global dimension of the sexual functioning. The domains follow the characteristics: desire (2 items); arousal (4 items); lubrication (4 items); orgasm (3 items); satisfaction (3 items); and pain (3 items).

The items are provided in a *Likert* scale varying from 1 to 5 in the items 1, 2, 15 and 16; and varying from 0 to 5 in the items 3, 4, 5, 6, 7, 8, 9, 10, 11, 12, 13, 14, 17, 18 and 19. The score can vary from 2 to 36, and the low scores are associated to a worse sexual functioning, while the highest scores are associated with better responses of sexual functioning. In the present study, scores ≤26.5 points are considered as moderating factors of the presence or absence of sexual dysfunction, according to previous studies.^13^ ^14^ ^15^

### Statistical Analysis

Scores from the FPI-II were obtained by the sum of raw data extracted from the thirteen characteristics and transformed afterwards in a percentile of each protocol according to the personality inventory manual.^10^ Analyzing each protocol, the test brings thirteen characteristics of primary order and three characteristics of second order, enabling the classification of the most notable personality traits.

The FSFI was considered a quantitative variable (when the average of its results on the assessed group was used), and as a qualitative variable (when it was categorized in women “with sexual dysfunction” and “without sexual dysfunction”). An analysis of the psychometric properties and internal consistency from the responses obtained with the FPI-II was also conducted, as well as from the FSFI instrument using the Cronbach Alpha coefficient, with cut-off point above α ≥0.70. This characterizes the instrument with good internal consistency, according to the literature.^16^

Next, the extracted data was tabulated into data sheets in the software Microsoft Excel 2007 (Microsoft Corporation, Redmond, WA, EUA). For statistical analysis, the qualitative variables of absolute frequency and relative frequency were used, and considered the quantitative variables by median, with a percentile of 25 and 75 and a confidence interval (CI) of 95% of the median, since the data did not present normal distribution tested by the Shapiro-Wilk test, (*p *< 0.05).

The Mann-Whitney test was also used (with confidence levels of 95%; with *p* < 0.05) to analyze the association between sexual self-perception and sexual dysfunction, with the variables age and personality traits from the FPI-II and the FSFI. The chi-squared test was used to analyze the association between qualitative variables.

Multivariate Poisson regression adjusted for robust variance was used to analyze the factors associated with the prevalence of sexual dysfunction by FSFI. The level of significance was 5%. Finally, the software Stata version 11.0 (StataCorp, College Station, TX, USA) was used for the statistical analyses.

The software PS Power and Sample Size (Vandebilt University, Nashville, TN, USA),^17^ was used to compute the sample statistical power, which was used to detect differences between the observed scores for affective needs and for organization needs (data with significance value), showed a power of 91.1% and 74.9%, respectively, considering the probability of type 1 (α) error as 5%.

## Results

Two hundred women were invited to participate as subjects in the present research. After reading the informed consent form, 128 women did not accept to participate; 72 accepted to participate and signed the informed consent form. After the validation of the inclusion and exclusion criteria, only 43 women were included in the final analyses; 29 women were not included due to non-answered questionnaires; non-completed tests; or for being under hormonal replacement therapy. From this screening, 2 groups were formed: 21 women with poor sexual self-perception (group I) and 22 women with good sexual self-perception (group II).

It is worth highlighting that the median age of the patients did not show any difference in the comparison between the poor sexual self-perception group (52 years; CI 49.4–57.0), and the good sexual self-perception group (54 years; CI 51–57). On the other hand, having or not completed high school showed an impact on sexual perception (*p* = 0.004), as shown in Table 1.

**Table 1:** Variables distribution according to the screening questionnaire, based on self-reporting by the patients

Variables	Poor sexual self-perception *n* =21	Good sexual self-perception *n* = 22	
	Median (95% CI)		p-value*
Age	52 (49.4–57.0)	54 (51–57.0)	0.542
BDI	9 (7–22.0)	13 (6–16.1)	0.679
FSFI	15 (9.9–20.5)	22.9 (16.4–27.1)	0.025
FPI—N. Control^I^	60 (45–70)	65 (44.3–85)	0.550
FPI—N. Affective^II^	5 (5–5)	57.5 (34.3–85)	< 0.01
FPI—N. Organization^III^	5 (5–5)	27.5 (9.6–65.3)	< 0.01
	***n*** ** (%)**		p-value**
Complete high school			
No	16 (69.6)	7 (30.4)	0.004
Yes	5 (25)	15 (75)	
Paid work			
No	15 (57.7)	11 (42.3)	0.151
Yes	6 (35.3)	11 (64.7)	

Abbreviations: BDI, Beck depression inventory; CI, confidence interval; FPI, factorial personality inventory; FSFI, female sexual function index.

*p*-value* Mann-Whitney test (CI: 95%).

*p*-value**: Chi-square test; I: Control and opposition necessity; II: Affective necessity; III: Organization necessity.

The answer to the BDI did not show any difference between the two groups, but both showed CIs indicating absence and presence of depressive symptoms. The average score on the FSFI showed a difference between the two groups, 15 points in the poor sexual self-perception group and 22.9 points in women who considered having a good sexual life. However, considering the CIs, both groups showed women with sexual dysfunction, according to Table 1.

Considering the division according to their sexual self-perception, women with poorer sexual self-perception showed fewer personality traits from affective and organization necessity types, compared with the women reporting good sexual self-perception (Table 1).

In Table 2, it is possible to observe the group division of women with or without sexual dysfunction, according to the FSFI. In this division, the results obtained by the BDI did not show any significant difference between the groups. Likewise, the control and opposition, affective and organization types of personality necessities did not show any significant difference among groups.

**Table 2:** Personality traits considering sexual dysfunction according to the FSFI

Variables	With sexual dysfunction *n* = 34	No sexual dysfunction *n* = 9	*p-value* d
	Median (95% CI)		
Age	53.5 (50–56.4)	51 (51–56.9)	0.976
BDI	14 (7.7–20.2)	6 (2–15.7)	0.063
N. Contr. Opposition^a^	65 (48.8–71.1)	50 (5–85)	0.401
N. Affective^b^	5 (5–56.1)	40 (15.7–70)	0.273
N. Organization^c^	5 (5–25)	15 (5–62.2)	0.782
	**n (%)**		***p-value*** e
Complete high school			
No	20 (87.0)	3 (13.0)	0.173
Yes	14 (70.0)	6 (30)	
Paid work			
No	22 (84.6)	4 (15.4)	0.269
Yes	12 (70.6)	5 (29.4)	

Abbreviations: BDI, Beck depression inventory; CI, confidence interval; FSFI, female sexual function index.

a Control and opposition necessity.

b Affective necessity

c Organization necessity

d *p*-value: - Mann-Whitney test (CI: 95%).

e *p*-value**:** Chi-square test.

In the multivariate analysis, it was observed that the BDI and the organizational need were not significant for the characterization of the sample (prevalence ratio [PR] = 1.01, *p* = 0.062; and PR = 1.00, *p* = 0.103, respectively); the sexual perception is associated with a higher prevalence of sexual dysfunction (PR = 0.61, *p* = 0.012), according to Table 3.

**Table 3:** Multivariate regression to analyze the factors associated with the prevalence of sexual dysfunction in postmenopausal women

Variables	PR (95% CI)	*p*-value
BDI	1.01 (0.99–1.03)	0.062
Organization need	1.00 (0.99–1.01)	0.103
Sexual perception	0.61 (0.42–0.89)	**0.012**

Abbreviations: BDI, Beck depression inventory; CI, confidence interval; PR, prevalence ratio.

*p*-value: Poisson regression with robust variance.

## Discussion

The results show an association between personality traits and sexual dysfunction in postmenopausal women when assessed by their sexual self-perception. Women with poor sexual self-perception showed fewer types of personality traits, such as affection and organization (FPI-II). A low level of education has also been shown to be significant for sexual dysfunction in relation to the self-report of the participants.

The evaluation of female sexual function is complex and there is no ideal methodology. The perception test used in the present study, besides being consecrated by a previous clinical trial by Davis et al,^7^ shows to be a simple and objective tool for the identification of sexual dysfunction. There are five direct, easy-to-understand questions. These criteria were consistent with the definition of hypoactive sexual desire disorder, following the criteria of the Diagnostic and Statistical Manual of Mental Disorders, fourth edition (DSM-IV). In the regression analysis, self-report was also positive for complaints of sexual dysfunction. The FSFI, despite being one of the main tools for evaluating sexual function, is criticized for its complexity.^18^ ^19^ ^20^ ^21^ ^22^

In the present study, age and depressive symptoms did not have any influence on the perception of the women of their sexual life. Likewise, differences on these variables were not found when the subjects were classified according to their FSFI scores. It is observed that, according to the BDI scores, women with sexual dysfunction have a tendency to show more depressive symptoms.

The researched literature points out to the fact that depressive symptoms are prevalent in women in the transition to menopause or during the climacteric, with the occurrence varying from 26^1^ to 60% of the women in this phase.^18^

The personality traits of affective and organization necessities showed differences between the good and poor sexual self-perception groups. The group with poor sexual self-perception showed fewer characteristics from both personalities when compared with the good sexual self-perception group. However, when sexual dysfunction was measured by the FSFI scores, the groups were similar according to all personality traits.

The most important specific personality traits in affective necessity are:

- **Affiliation:** difficulty to relate affectively and to self-maintain faithful to somebody else, making friends, maintain them or become intimate with somebody;
- **Assistance:** difficulty to assist and treat people with compassion and tenderness;
- **Gentle:** difficulty in searching for help, protection, consolation or forgiveness of others;
- **Intraception:** can let oneself be carried away by feelings and diffuse tendencies, showing more difficulty in conducting one's own life and in making judgments based on concrete facts of reality;
- **Deference:** difficulty in admiring, prestige, supporting, honoring, praising, imitating hierarchically superior people, accepting superior orders and conforming to customs and traditions;
- **Change:** difficulty in changing, upon one's own effort, certain situations or certain personal characteristics.

In the literature, personality traits, like affective necessities, are understood as positive factors to a better sex and life quality for postmenopausal women.^7^ ^19^

After analyzing the orgasmic capacity in postmenopausal healthy women without hormonal replacement treatment, Penteado et al^19^ concluded that the orgasmic capacity is positively associated to more affective relationships with sexual partners, supporting the results found by the present research.

From the present study, it can be inferred that affectivity shows itself in two dimensions: the first is positive, because it reflects how much a person feels enthusiastic, active and alert; the second is negative, because it is related to anguish, to dissatisfaction, to aversive states of mood, to anger, to guilt, to grief, and to fear. Women with a low level of affectivity have the tendency to relate affection to a negative connotation, evoking feelings and characteristics of neuroticism. This one is referred to the chronification of adjustment and emotional instability, patterns associated with psychological discomfort and resulting cognitive-behavioral styles.^20^

Affection as a personality trait has been considered a protection factor, so that people do not live intensely emotional suffering, do not develop anxiety symptoms, low self-esteem, and do not experience more impulsivity symptoms.^21^ The critical thinking around this concept can indicate the possibility that, probably, women with good sexual self-perception in their psychological and emotional development lived more affective traits in their social and familial environment than the ones with poor sexual self-perception. In regard to the necessity of organization characteristics, for being one specific group of personality assessed by the FPI-II, the authors did not find any studies in the scientific literature to compare the resulting data. In this group of psychological needs, women with poor sexual self-perception showed less personality traits in:

- **Persistence**: difficulty to intensely dedicate themselves to one task until it is finished, even if, for that, it is necessary to disrespect their own limits;
- **Order**: difficulty to maintain order and to value cleaning, the balance, and the precision from objects of the exterior world;
- **Performance**: difficulty in overcoming obstacles, achieving difficult actions, and executing tasks independently and with maximum quickness.

The necessity of organization, such as an expression of one's own personality, when not developed, tends to reflect in the way women live daily-life peculiarities, which, in the present study, points to difficulties in the sexual life. It is worth mentioning that women with good sexual perception had median scores on these characteristics, which suggests a higher flexibility and dynamism to deal with issues related to productivity, commitment in achieving their goals and non-fulfillment of their own wishes, which also suggests a good capacity to deal with issues in their sexual lives.

Nonetheless, it is important to point out that, after the group division of women according to the obtained FSFI scores, the groups did not show significant differences. The main reason for this and a vulnerability of the present study is that, after the group division, only nine women presented healthy sexual functioning. Therefore, the CI referring to the personality inventory score became wide in the three categories and did not allow a clinical characterization on the personality of this group. This result does not disapprove the other results found in the valid classification on self-perception but impose the necessity of a new study with adequate number of women for evaluation by the FSFI.

As closing remarks, it is important to highlight that studies associating personality traits and sexual dysfunction in postmenopausal women based on the scores obtained with the FPI-II are scarce in the scientific literature. The present study proposes to encourage the discussion and knowledge about the influence of psychological factors on the sexuality of middle-aged women.

## Conclusion

Finally, in the present study, postmenopausal women with poorer sexual self-perception demonstrated personality traits with low affective and organization necessities. The low level of schooling was also significant for the perception of sexual dysfunction. New researches addressing personality aspects of postmenopausal women are necessary for the understanding of its impact in sexual dysfunction, and, therefore, enable an adequate therapeutic approach.
